# Immunopathogenesis of Chlamydial Pelvic Inflammatory Disease: The Role of Heat-Shock Proteins

**DOI:** 10.1155/S1064744994000475

**Published:** 1994

**Authors:** Jorma Paavonen, Matti Lehtinen

**Affiliations:** 1 Department of Obstetrics and Gynecology University of Helsinki Helsinki Finland; 2 Department of Chronic Viral Diseases National Public Health Institute Helsinki Finland


Infectious Diseases in Obstetrics and Gynecology 2:105–110 (1994)
© 1994 Wiley-Liss, Inc.

### Immunopathogenesis of Chlamydial Pelvic Inflammatory Disease: The Role of Heat-Shock Proteins

Pelvic inflammatory disease (PID) is the most important complication of sexually transmitted chlamydial infections, causing major medical, social, and economic problems. PID is an ascending infection, with well over half of the cases caused by *Chlamydia trachomatis* (CT), *Neisseria gonorrhoeae* (GC), or both. However, other microorganisms representing abnormal vaginal flora, such as those present in bacterial vaginosis, are also commonly involved.[1,2] GC rates have rapidly decreased in most developed countries, while CT rates have remained high. Thus, the relative role of CT in the etiology of PID has increased. In the 1980s, it also became obvious that the clinical spectrum of PID manifestations was changing. An increasing proportion of cases were atypical or silent, and the typical "textbook" PID was becoming a rare disease. Subsequently, there has been a dramatic drop in the incidence of inpatient PID. Clearly, this change reflects a change in the etiologic pattern of PID.

A large number of seroepidemiologic studies support the concept of silent PID by demonstrating a strong link between serum antibodies to CT and tubal factor infertility or ectopic pregnancy in patients with or without histories of PID.[3] Concern about the problem of unrecognized PID has also led to a fundamental change in the recommendations for PID diagnosis. The following set of simple, easily ascertained minimum clinical criteria should trigger antibiotic treatment for probable PID: lower abdominal tenderness; bilateral adnexal tenderness; cervical motion tenderness; no evidence of a competing diagnosis, e.g., positive pregnancy test, acute appendicitis.[4] This important change in direction, from a laboratory- and laparoscopy-based diagnosis toward a syndromic diagnosis, should increase sensitivity by decreasing the false-negative rate. Although the more sensitive approach will result in some unnecessary antibiotic treatment, it will also lead to the provision of proper therapy earlier in the course of PID. Delay of care, as well as unrecognized PID, is an important cause of impaired fertility, particularly in chlamydial PID.[5]

Worldwide, the magnitude of PID-related morbidity is still enormous. The proportion of tubal factor infertility of all infertility ranges from 37% in developed countries to 85% in developing countries.[6] Our understanding of the pathogenesis of PID and PID-related sequelae has increased along with a better understanding of the immunopathogenesis of CT infection. Early studies had already suggested that a single acute infection per se could not account for all the pathology associated with chlamydial disease.[7] The persistence of chronic inflammation after proper therapy for chlamydial infection has been a puzzling phenomenon in many clinical studies. For instance, persistent cervicitis not explained by relapse or reinfection was found 3 months after therapy in 23–33% of women treated for chlamydial mucopurulent cervicitis.[8] Similarly, not only adhesion formation and tubal occlusions but also persistent inflammation of the fallopian tubes in the absence of CT were frequently seen during a second-look laparoscopy performed 4–6 months after the index episode.[9]

Received August 8, 1994
Accepted August 11, 1994

Editorial

The main sources of a repeat or prolonged antigenic stimulus leading to systemic sensitization against chlamydiae are the persistence of subclinical infection after unsatisfactory treatment and recurrent infection. Subclinical PID (plasma-cell endometritis) has been detected in two-thirds of women with chlamydial mucopurulent cervicitis.[2,10] Poor compliance with antibiotic therapy is a major problem, increasing the risk of persistent infection.[11] In a survey of general practitioners in Norway, 63 different regimens were used for the treatment of PID. Only 46% of these regimens were compatible with standard treatment guidelines.[12] Similarly, recurrent infections are extremely common, particularly among adolescents.[13,14]

CT is a strong immunogen that stimulates both humoral and cell-mediated immune responses.[15] This organism can induce perturbations in immune function that may assist its survival in an infected host.[16] The ability of CT to convert from resting to replicating infectious forms within host cells creates increasing difficulties in eliminating this microbe. If the first line of immune surveillance against CT fails (Langerhans cells, macrophages, naturally occurring or specific secretory antibodies), the invasion of chlamydiae results in infection of epithelial cells and fibroblasts. Infected cells release chlamydiae, and increasing numbers of these organisms enter lymphoid tissue, a situation characterized by striking polyclonal B-cell hyperplasia.[2,10,17,18] The release of large quantities of chlamydial particles from infected cells leads to a perturbation of the immune response. Our recent findings suggest that in moderate plasma-cell endometritis, proliferating T cells stimulate a specific antibody response, but in severe endometritis, they downregulate [type-1 helper T cell (Th1-type) response] or upregulate (Th2-type response) polyclonal antibody production (Lehtinen M, Rantala I, Aine R, Paavonen J, unpublished data, 1994). Thus, the indication of a Th1- or Th2-type response may be an important determinant in chlamydial disease pathogenesis. Of the proliferating T cells, Th1 cells regulate classic delayed hypersensitivity (DHS) reactions. The cytokine profile of a type-1 helper pathway (dominant cell-mediated response) is different from that of a type-2 helper pathway (dominant antibody response). Interferon (IFN)-gamma and interleukin 2, in particular, play major roles in turning on the efferent arm of cell-mediated immunity. High levels of IFN-gamma have been detected in endocervical secretions of patients with chlamydial cervicitis[19] and in sera of patients with PID.[20] However, in vitro studies have shown that IFN-gamma mediates the development of atypical morphologically enlarged chlamydial forms. Such forms display greatly reduced levels of outer-membrane proteins such as MOMP, 60-kD OMP, and lipopolysaccharide, but a continued production of heat-shock protein (HSP) 60.[21,22] If IFN-gamma induces similar abnormal persistent organisms in vivo, such persistently infected cells could augment the pathogenesis of the chronic chlamydial sequelae that follow chlamydial infection by serving as depots of antigen capable of stimulating chronic inflammation.

We now know that the chronic sequelae of chlamydial infection are caused by a DHS reaction to chlamydial HSPs, particularly the 57-kD HSP. HSPs serve as important antigens of infectious agents and are among the most conserved molecules in phylogeny.[23] The predominant chlamydial stress proteins belong to the 12-kD (or GroES-like) HSP family, 60-kD (or GroEL-like) HSP family (HSP60), and 70-kD (or DnaK-like) HSP family (HSP70). The amino acid homology of the HSPs with their microbial or human counterparts is high, varying from 42 to 90% (Table 1).[24–27]

A number of studies have looked at the antibody responses to HSPs induced by

**TABLE 1. Amino acid sequence-deduced homology of *C. trachomatis* L2 HSPs with microbial and human GroES, GroEL, and DnaK analogs[a]**

| Organism | GroES (%) | GroEL (%) | DnaK (%) |
|---|---|---|---|
| *Chlamydia trachomatis D* | — | — | 94 |
| *Chlamydia pneumoniae* | 89 | 95 | 87[b] |
| *Chlamydia psittaci* | 85 | 93 | — |
| *Coxiella burnetii* | 33 | 61 | — |
| *Bacillus megaterium* | — | — | 57 |
| *Escherichia coli* | 31 | 60 | 57 |
| *Mycobacterium tuberculosis* | 44 | 57 | — |
| Human | — | 48 | 42 |

[a]Data from references 24–27.
[b]*C. trachomatis D* vs. *C. pneumoniae*.

CT and generally found a good correction between serum antibodies to HSP60 and PID, tubal factor infertility, and ectopic pregnancy.[28–33] In women with chlamydial PID, a history of PID, cervicitis, or chlamydial infection; laparoscopically observed tubal obstruction; laparoscopically observed tubal inflammation; and the presence of moderate-to-severe adhesions were all associated with HSP60 antibodies.[31] A correlation of antibodies to HSP60 with microimmunofluorescence antibodies (toward chlamydial major outer-membrane protein antigen) has been detected in some,[31,33] but not all studies.[32]

The cell-mediated immune response to chlamydial HSPs has been less well studied, although it undoubtedly plays a critical role in the DHS response. The role of DHS in the pathogenesis of chlamydial salpingitis has been studied in the monkey "pocket" model.[34] Pigtailed monkeys were sensitized by inoculation of live CT organisms into subcutaneous pockets containing salpingeal autotransplants. When recombinant chlamydial HSP60 (rHSP60) was injected into pockets either previously sensitized or not sensitized in the same monkey, a typical DHS reaction was observed. Furthermore, a positive lymphocyte-proliferation response of peripheral-blood mononuclear cells to rHSP60 was more common in women with PID than in women without PID or in controls.[35] Interestingly, most of those with positive responses had histories of PID or ectopic pregnancy, suggesting that the duration of exposure is an important variable of the CT-specific T-cell response.

A molecular mimicry of human and chlamydial HSPs may play a role in autoimmunity. An autoimmune response can begin even if the molecular mimicry is not exact. The local accumulation of chlamydial HSPs and cross-reactive immune response can generate an autoimmune reaction that explains part of the inflammatory reaction and pathology seen after chlamydial PID. The presence of human GroEL-like stress protein in the upper genital tract has been demonstrated by immunohistochemistry using ML30 monoclonal antibody.[36] Therefore, chlamydial and human HSP60 are candidate targets to autoimmune T cells, although practically nothing is known of possible cross-reactive T-cell epitopes. Recent epitope scanning studies of chlamydial HSP60 have revealed distinct B-cell epitopes. Fifteen percent of infants with chlamydial pneumonia have shown low levels of autoantibodies to chlamydial and human peptide-defined epitopes with high amino acid homology.[37] However, although autoimmune reactions may play some role in the chronic sequelae of chlamydial infections, the DHS response associated with persistent or recurrent infections clearly plays a more important role.

In conclusion, tubal factor infertility and ectopic pregnancy are important public health problems. Understanding the interactions of chlamydiae with the immune system is of paramount importance in the prevention of chlamydial PID. Although chlamydial infections can be treated with antibiotics, chlamydiae have several properties that increase the risk for subclinical infections. These properties are based on immune perturbations induced by the organisms themselves. Not surprisingly, tertiary prevention of PID has largely been disappointing because substantial tubal damage has already occurred by the time any symptoms develop. Secondary prevention means preventing lower genital-tract infection from ascending to the upper genital tract. Health care and provider behaviors play a critical role in secondary prevention. Recent studies suggest that intervention trials with selective screening for chlamydial infections effectively reduce the incidence of PID.[38] Primary prevention involves preventing both exposure to and acquisition of chlamydial infection. Clinicians play an important role in primary prevention by questioning about high-risk sexual behavior, by encouraging screening tests for those at risk, by ensuring that male sexual partners are evaluated and treated, and by counseling about safer sexual practices. Fortunately, not all women with chlamydial infections develop a DHS reaction to the HSPs. Genetically determined autoimmune responses to the conserved epitopes of HSPs are probably relatively rare. However, studies of the fertility prognosis of asymptomatic women with chlamydial cervical infections in relation to HSP60 antibodies should be undertaken, and major T-cell epitopes of HSPs should be defined to find out whether specific epitopes are responsible for the stimulation of type-1 and type-2 T-helper cells or autoimmune responses.

*Jorma Paavonen, M.D.*
*Department of Obstetrics and Gynecology*
*University of Helsinki*

*Matti Lehtinen, M.D.*
*Department of Chronic Viral Diseases*
*National Public Health Institute*
*Helsinki, Finland*

### REFERENCES

1. McCormack WM: Pelvic inflammatory disease. N Engl J Med 330:115–119, 1994.
2. Paavonen J, Teisala K, Heinonen PK, et al: Microbiological and histopathological findings in acute pelvic inflammatory disease. Br J Obstet Gynaecol 94:454–460, 1987.
3. Cates W, Wasserheit JN: Genital chlamydial infections: Epidemiology and reproductive sequelae. Am J Obstet Gynecol 164:1771–1781, 1991.
4. Rolfs RT: Think PID. New directions in prevention and management of pelvic inflammatory disease. Sex Transm Dis 18:131–132, 1991.
5. Hillis SD, Joesoef R, Marchbanks PA, Wasserheit JN, Cates W, Weström L: Delayed care of pelvic inflammatory disease as a risk factor for impaired fertility. Am J Obstet Gynecol 168:1503–1509, 1993.
6. World Health Organization: Infections, pregnancies, and infertility: Perspectives on prevention. Fertil Steril 47:964–968, 1987.
7. Schachter J: Pathogenesis of chlamydial infections. Pathol Immunopathol Res 8:206–20, 1989.
8. Paavonen J, Roberts PL, Stevens CE, et al: Randomized treatment of mucopurulent cervicitis with doxycycline or amoxicillin. Am J Obstet Gynecol 161:128–135, 1989.

9. Teisala K, Heinonen PK, Aine R, Punnonen R, Paavonen J: Second laparoscopy after treatment of acute pelvic inflammatory disease. Obstet Gynecol 69:343–346, 1987.
10. Paavonen J, Aine R, Teisala K, et al: Chlamydia endometritis. J Clin Pathol 38:726–732, 1985.
11. Katz BP, Zwicki BW, Caine VA, Jones RB: Compliance with antibiotic therapy for *Chlamydia trachomatis* and *Neisseria gonorrhoeae*. Sex Transm Dis 19:351–354, 1992.
12. Aavitsland P: Survey of the treatment of *Chlamydia trachomatis* infection of the female genital tract. Acta Obstet Gynaecol Scand 71:356–360, 1992.
13. Jones RB: Treatment of *Chlamydia trachomatis* infections of the urogenital tract. In Bowie WR et al. (eds): Proceedings of the Seventh International Symposium on Human Chlamydial Infections, Harrison Hot Springs, British Columbia, June 24–29, pp 509–518, 1990.
14. Hillis SD, Nakashima A, Marchbanks PA, Addiss DG, Davis JP: Risk for recurrent *Chlamydia trachomatis* infections in women. Am J Obstet Gynecol 170:801–806, 1994.
15. Lehtinen M, Paavonen J: Heat-shock proteins in the immunopathogenesis of chlamydial pelvic inflammatory disease. In Orfila J et al. (eds): Chlamydial infections. Proceedings of the Eighth International Symposium of Human Chlamydial Infections, Chantilly, Societa Editrice Esculapio, pp 599–610, 1994.
16. Räsänen L, Lehtinen M, Lehto M, Paavonen J, Leinikki P: Stimulation of umbilical cord blood lymphocytes by *Chlamydia trachomatis*. Infect Immun 54:28–31, 1986.
17. Kiviat NB, Wölner-Hanssen P, Eschenbach DA, et al: Endometrial histopathology in patients with culture-proved upper genital tract infection and laparoscopically diagnosed acute salpingitis. Am J Surg Pathol 14:167–175, 1990.
18. Kiviat N, Paavonen J, Wölnér-Hanssen P, et al: Histopathology of endocervical infection caused by *Chlamydia trachomatis*, herpes simplex virus, *Trichomonas vaginalis*, and *Neisseria gonorrhoeae*. Hum Pathol 21:831–837, 1990.
19. Arno JN, Ricker VA, Battgeier BE, Katz BP, Caine VA, Jones RB: Interferon-gamma in endocervical secretions of women infected with *Chlamydia trachomatis*. J Infect Dis 162:1385–1389, 1990.
20. Grifo JA, Jeremia J, Ledger WJ, Witkin SS: Interferon-gamma in the diagnosis and pathogenesis of pelvic inflammatory disease. Am J Obstet Gynecol 160:26–31, 1989.
21. Beatty PR, Stephens RS: Identification of *Chlamydia trachomatis* antigens by use of murine T cell lines. Infect Immun 60:4598–4603, 1992.
22. Beatty WL, Byrne GI, Morrison RP: Morphologic and antigenic characterization of interferon gamma-mediated persistent *Chlamydia trachomatis* infection in vitro. Proc Natl Acad Sci USA 90:3998–4002, 1993.
23. Kaufmann SHE: *Heat shock proteins and the immune response. ImmunolToday 11:129–136,* 1990.
24. Birkelund S, Lundemose AG, Christensen G: The 75-kilodalton cytoplasmic *Chlamydia trachomatis* L2 polypeptide is a DnaK-like protein. Infect Immun 58:2098–2104, 1990.
25. Cerrone MC, Jeffrey JMA, Stephens RS: Cloning and sequencing of the gene for heat shock protein 60 from *Chlamydia trachomatis* and immunological reactivity of the protein. Infect Immun 59:79–90, 1991.
26. Kikuta LC, Puolakkainen M, Kuo GC, Campbell LA: Isolation and sequence analysis of the *C. pneumoniae* GroE operon. Infect Immun 59:4665–4669, 1991.
27. Kornak JM, Kuo GC, Campbell LA: Sequence analysis of the gene encoding the *Chlamydia pneumoniae* DnaK protein homolog. Infect Immun 59:721–725, 1991.
28. Brunham RC, Peeling R, Maclean I, McDowell J, Pusson K, Osser S: Postabortal *Chlamydia trachomatis* salpingitis: Correlating risk with antigen specific serologic responses and with neutralization. J Infect Dis 155749–755, 1987.
29. Brunham RC, Peeling R, Maclean I, Koseim ML, Paraskevas M: *Chlamydia trachomatis* associated ectopic pregnancy: Serologic and histologic correlates. J Infect Dis 165:1076–1081, 1992.
30. Peeling RW, Toye B, Jessamine P, Claman P, Gemmill I, Sankar P: Antibody response to the chlamydial HSP60 (Chsp60) in different populations. Sex Transm Dis 18:131–132, 1994.
31. Stamm WE, Peeling RW, Money D, et al: Prevalence and correlates of antibodies to chlamydial HSP60 in *C. trachomatis* infected women. In Orfila J et al. (eds): Chlamydial Infections. Proceedings of the Eighth International Symposium of Human Chlamydial Infections, Chantilly, Societa Editrice Esculapio, pp 614–617, 1994.
32. Toye B, Laferriére C, Claman P, Jessamine P, Peeling R: Association between antibody to the chlamydial heat-shock protein and tubal infertility. J Infect Dis 168:1236–1240, 1993.

33. Wagar EA, Schachter J, Bavoli P, Stephens RC: Differential human serologic response to two 60.000 molecular weight *Chlamydia trachomatis* antigens. J Infect Dis 162:922–927, 1990.
34. Patton DL, Cosgrowe Sweeney YT, Kuo C-C: Demonstration of delayed hypersensitivity in *Chlamydia trachomatis* salpingitis in monkeys: A pathogenic mechanism of tubal damage. J Infect Dis 169:680–683, 1994.
35. Witkin SS, Jeremias J, Toth M, Ledger WJ: Cell-mediated immune response to the recombinant 57-kDa heat-shock protein of *Chlamydia trachomatis* in women with salpingitis. J Infect Dis 167:1379–1383, 1993.
36. Evans DJ, Norton P, Ivany J: Distribution in tissue sections of the human GroEL stress-protein homologue. APMIS 98:437–441, 1990.
37. Paavonen J, Lähdeaho ML, Puolakkainen M, Mäki M, Parkkonen P, Lehtinen M: Antibody response to B cell epitopes of *Chlamydia trachomatis* 60 kDa heat shock protein and corresponding mycobacterial and human peptides in infants with chlamydial pneumonitis. J Infect Dis 169:908–911, 1994.
38. Scholes D, Stergachis A, Heidrich FA, et al.: Selective screening for chlamydia reduces the incidence of pelvic inflammatory disease: Results from a randomized intervention trial. In Orfila J et al. (eds): Chlamydial Infections. Proceedings of the Eighth International Symposium of Human Chlamydial Infections, Chantilly, Societa Editrice Esculapio, pp 28–31, 1994.
